# In-Hospital Mortality in Patients With Acute Myocardial Infarction: A Literature Overview

**DOI:** 10.7759/cureus.66729

**Published:** 2024-08-12

**Authors:** Khalid A Alnemer

**Affiliations:** 1 Department of Internal Medicine, College of Medicine, Imam Mohammad Ibn Saud Islamic University, Riyadh, SAU

**Keywords:** clinical factors, biomarkers, non-st-elevation myocardial infarction, st-elevation myocardial infarction, in-hospital mortality, acute myocardial infarction

## Abstract

Acute myocardial infarction (AMI) continues to be a predominant cause of global morbidity and mortality, with in-hospital mortality (IHM) serving as a pivotal metric for patient outcomes. This review explores the influence of several clinical variables on IHM in individuals with AMI. Factors such as age, gender, body mass index (BMI), smoking habits, existing comorbidities, prior coronary artery bypass graft (CABG), percutaneous coronary intervention (PCI), and biomarkers, including high-sensitivity cardiac troponin T (hs-cTnT) and creatine kinase MB (CK-MB), significantly affect the prognosis of the patient. Advanced age and comorbid conditions such as diabetes and hypertension exacerbate myocardial damage and systemic impacts, thus increasing IHM. Gender and BMI are also critical, and women and patients with obesity face different risks. Smoking increases both the risk of AMI and IHM, underscoring the importance of cessation interventions. ST-elevation myocardial infarction is associated with elevated IHM and requires immediate reperfusion therapy, while non-ST-elevation myocardial infarction requires customized management for risk assessment. Previous CABG and PCI add complexity to AMI treatment and elevate IHM due to pre-existing coronary pathology and the intricacies of the procedures involved. The application of biomarker-centered techniques facilitates the swift identification of individuals at elevated risk, improves therapeutic planning, and reduces IHM for patients with AMI. Understanding and incorporating these clinical determinants are essential to optimize the management of AMI, minimize IHM, and improve patient outcomes. This all-encompassing strategy requires ongoing research, quality improvement efforts, and personalized care approaches.

## Introduction and background

Acute coronary syndrome (ACS) represents a common clinical manifestation of cardiovascular disease (CVD), which annually results in a large number of hospital admissions and emergency department consultations around the world [1]. Despite advances in reperfusion strategies and improvements in supportive pharmacological treatments, people with acute myocardial infarction (AMI) continue to experience a significant likelihood of subsequent cardiovascular events and mortality rates (8.9 million deaths worldwide in 2019) that are noteworthy [1-3]. Consequently, many risk determinants and prognostic indices have been established to forecast short- and long-term detrimental consequences [4].

The prevalence of AMI or ACS in the elderly has decreased significantly due to improvements in both primary and secondary CVD prevention methods. However, a similar decline is not evident among younger demographics, as defined by Ando et al. [5]. Despite the advances in pharmacological treatment and cardiac catheterization techniques, the rate of in-hospital mortality (IHM) for those with AMI remains an area fraught with clinical complexities. It is imperative to discern the clinical determinants related to IHM within this group. This knowledge is vital for practical risk assessment, prognosis determination, and design of interventions designed to improve patient survival rates; these points were critically analyzed [6].

As innovative approaches to managing AMI (such as fibrinolytic and invasive therapies) emerge, frameworks to assess mortality risk also advance. In recent times, numerous models have been established to measure mortality risk among patients with myocardial infarction (MI), which includes impairment in diastolic and systolic function, atherosclerotic disease, and coronary artery anomalies [7]. These models consider immediate (during hospitalization) and extended (across various intervals after discharge from medical facilities) outcomes. Among these are clinical scoring instruments specifically designed for application in hospital settings, offering cardiologists practical tools for day-to-day use in clinical settings [8].

Primary percutaneous coronary intervention (PCI) is recognized as the superior method of reperfusion treatment for AMI, particularly in cases where patients have ST-segment elevation myocardial infarction (STEMI). However, research focusing on younger demographics needs to be more varied due to their relatively low rates of AMI manifestation [5]. The European Society of Cardiology (ESC) endorses clinical scales that assess mortality risks among MI sufferers within its most recent directives on MI management. Currently, the GRACE score is valued for its exceptional discriminatory capacity. Thus, it is the fundamental index for evaluating individuals with NSTEMI (non-ST-elevation myocardial infarction) [9]. Observational studies from the United States and Canada have documented a substantial decrease in IHM rates among patients with AMI who received PCI intervention compared to those who did not [10]. For clinicians, risk evaluation metrics play an integral role by enhancing clinical judgment processes, informing about appropriate pharmacological treatments, helping to determine the necessary lengths of hospital stays, and helping to craft follow-up care after discharge strategies [8].

When examining contemporary methodologies to assess mortality risk in patients with AMI, it becomes evident that they predominantly use a consistent array of factors. These include initial clinical challenges, vital statistics observed upon hospital admission, laboratory test results, and presentation of MI as seen on electrocardiograms (ECG), all gauged during the initial patient encounter. By analyzing these variables, healthcare professionals can determine the preliminary mortality risk for those hospitalized with confirmed MI. At the heart of the management of AMI lies a comprehensive grasp and synthesis of various clinical indicators that shape patient outcomes, delineate risk categories, and influence IHM rates.

Our current review of the literature investigates the principal clinical determinants linked to IHM among individuals diagnosed with AMI. The probability of survival after an AMI diagnosis differs markedly between the demographics of the patients due to a multitude of factors, such as gender variance, ethnic background, chronological age, specific types of AMI, pre-existing health conditions, and chosen medical interventions [10]. Our goal is to integrate existing clinical research findings and authoritative protocols to provide an exhaustive analysis of prognostic indicators of IHM and their implications for evaluating risk and guiding treatment choices. By elucidating these elements, we intend to deepen our understanding of the intricate mechanisms that influence mortality associated with AMI and contribute insights conducive to refining patient care delivery while striving toward decreasing death rates.

## Review

Clinical factors associated with IHM in patients with AMI

Effective handling of AMI requires a thorough understanding and synthesis of multiple clinical elements such as age, gender, co-morbidities (like hypertension (HTN) and diabetes), body mass index (BMI), biological markers, etc. These collective elements dictate the patient's prognosis, risk assessment, and treatment results, underscoring the need for a multidisciplinary method and personalized care tactics to improve results and minimize hospital mortality in patients with AMI.

Age

The crucial role of age in AMI-induced IHM cannot be understated, given its extensive influence on the patient's physiological state, the severity of comorbid conditions, and their response to treatment. It is well established that advanced age is a substantial standalone forecaster of negative results in patients with AMI.

With age, the cardiovascular system undergoes physiological transformations such as increased arterial rigidity, abnormal myocardial contractility and relaxation, and impaired coronary circulation [11]. Approximately 30-40% of all hospitalized patients with ACS are adults 75 years and older, and a significant proportion of ACS-related deaths are observed in this demographic within the United States [12,13]. Age is a significant risk factor for diffuse coronary disease and a worsening prognosis in hospitalized patients with unstable angina (UA) or NSTEMI [14]. The TIMI III registry revealed that patients 75 years and older with UA/NSTEMI exhibited more extensive and severe coronary disease, experiencing poorer outcomes both during hospitalization and within the first six weeks after discharge compared to those under 75 years of age [14].

Elderly individuals frequently exhibit an increased prevalence of concurrent medical conditions, including HTN, diabetes mellitus (DM), chronic renal disease, and peripheral vascular disease, which are recognized risk factors for cardiovascular events and IHM in patients with AMI [15]. In the same article, the coexistence of multiple comorbidities was reported to complicate the clinical management of AMI and increase the risk of IHM. Death rates have been documented to be higher in 55 and older patients compared to those under 55 years of age (OR: 4.07; 95% CI: 2.16-7.64) [15].

Elderly individuals may show unusual or vague signs of AMI, such as exhaustion, breathlessness, or disorientation, which could delay recognition and diagnosis of the critical incident [16]. The average duration from the appearance of symptoms to the initial medical interaction was recorded at 12.7 hours, with a range of 10 minutes to 96 hours. Increased the incidence of IHM [7]. Additionally, Khan et al. concluded that the non-standard manifestations of MI are extensive; patients may suffer from chest pain lacking the typical characteristics of angina pectoris or may not experience chest pain at all. Most of the patients were elderly and generally had pain and discomfort in the abdominal, cranial, and cervical areas [7]. Multiple research studies have established a strong link between advanced age and elevated IHM rates among patients with AMI with diabetes (OR: 2.33; 95%CI: 1.42-3.81; p= 0.001 [15], with heart failure of AMI at 25.9% [17]. Elderly subjects (about 6.3% above age 80 yrs) show higher short-term and long-term mortality rates after AMI compared to their younger counterparts, where IHM was associated with hypoxia at admission (OR: 1.70; 05%CI: 1.30-2.22) [18]. There was a notable association between age-adolescence and increased mortality rates among patients with AMI (P = 0.001) [15]. Older adults often suffer from chronic low-grade inflammation, which is associated with frailty and CVD [13]. A person who is frail is characterized by a physiological decline across multiple organ systems which increases vulnerability to stressors, increasing the likelihood of functional decline, hospitalization, and death [19]. In patients with frailty, less aggressive approaches may yield better outcomes due to increased risk for adverse outcomes. Clinical practitioners are advised to manage competing noncardiac risks in frail patients with MI by paying more attention [19].

Gender

In the realm of AMI and IHM, gender is a significant clinical variable that influences patient presentation, treatment approaches, and eventual outcomes. Historically, men have been perceived to be more susceptible to AMI than women, especially in their younger years [20]. On the contrary, women tend to experience AMI later in life (57± 7 years), often accompanied by nontraditional symptoms, which could result in missed diagnoses and treatment delays [20,21]. A 2022 study by Rohani et al. revealed that 18.8% (n = 105) passed away before discharge of all MI patients admitted to the hospital [15]. About 28.4% of these fatalities were women, while men accounted for 13.6%. When analyzing these groups, it was found that 88.7% (n = 93) of those who did not survive belonged to the age group 55 years or older.

The risk factors for AMI are differentially distributed between genders, leading to distinct patterns in its manifestation and prognosis. Women are more likely to suffer from comorbid conditions such as HTN, diabetes, and obesity. In contrast, men show a higher propensity toward habits such as smoking and excessive alcohol use [22,23]. It is hypothesized that estrogen confers cardio-protection, resulting in a lower incidence of AMI in premenopausal women compared to men of the same age [24]. However, this cardioprotective effect decreases after menopause, increasing the risk of AMI in older women. Asgari et al. found in their investigation that the majority (66.3%) of MI patients were male [25]. Rohini et al. observed that most of the hospitalized women (85%) were 55 years or older, while only 62% of the men belonged to this age group [15]. The literature reveals gender-based discrepancies in AMI management, with women receiving recommended treatments such as aspirin, beta-blockers, and reperfusion therapy less frequently. These disparities could potentially lead to poorer outcomes and higher mortality rates among women [23]. Numerous studies indicate elevated mortality rates in the hospital and 30 days after MI in women compared to men [26]. This trend is particularly evident among younger women (under 55), with the gender disparity decreasing with age [26]. For example, research by Nazzal and Alonso revealed a high IHM rate in women under 45 years of age (OR: 2.3; 95%CI: 1.5-3.3) [27]. The increase in early mortality in females is believed to be due to differences in the presentation of symptoms [28,29].

Elgendy et al. identified that, compared to their male counterparts, women who experienced AMI accompanied by cardiogenic shock received guideline-based treatment less frequently within the initial 24 hours and at the time of discharge [30]. This gender-based discrepancy has previously been observed in patients with AMI, regardless of the presence of cardiogenic shock [31]. Previous studies have confirmed that women are less likely to undergo cardiac catheterization and receive mechanical circulatory support devices [32]. On a positive note, the research did not reveal differences in the frequency of primary PCI among STEMI patients [32]. However, women were less likely to achieve a door-to-device time of less than 90 minutes [30]. The root of this delay remains unclear, whether it is attributable to patient-specific or system-wide delays in the recognition of STEMI (for example, due to gender-related variances in the presentation of symptoms or the ability of healthcare professionals to identify and appreciate symptoms) or whether it stems from post-STEMI recognition care is undetermined in the analysis by Elgendy et al. [28].

Smoking

Smoking is acknowledged as a contributing determinant of IHM in AMI scenarios, essentially accelerating the onset of STEMI in subjects supposedly healthier [33]. Although smoking plays a crucial role in the onset of atherosclerosis, numerous studies have indicated that smokers who receive fibrinolytic treatment for AMI exhibit better outcomes compared to non-smokers, as it causes thrombosis, arterial inflammation, and dilatation and dysfunction [34]. This phenomenon is called the "smoker paradox." For example, GUSTO I, the most extensive trial evaluating the impact of smoking on clinical outcomes, included 11,975 non-smokers, 11,117 former smokers, and 17,507 current smokers. Non-smokers showed significantly higher IHM (9.9% versus 3.7%) and 30-day mortality rates (10.3% versus 4.0%) [35]. The superior results observed in smokers after fibrinolysis can be attributable to several factors: first, smokers often have elevated levels of hematocrit and baseline fibrinogen, indicating a hypercoagulable state. These more active thrombogenic processes can lead to a more significant thrombus burden, which is more amenable to fibrinolytic therapy, resulting in higher patency rates and a higher probability of achieving TIMI-3 flow in the infarct-related artery after fibrinolysis [36]. Second, smokers exhibit a more favorable risk profile compared to non-smokers; they are generally younger (average age is 11 years younger in GUSTO I) and show a lower prevalence of diabetes, HTN, previous MI, and severe coronary artery disease [37].

A study by Song et al. found a decrease in IHM between current smokers and nonsmokers (OR: 0.78, 95% CI: 0.69 to 0.88, p=0.001) [38]. However, the same study did not observe significant differences in IHM between former smokers and non-smokers (OR: 0.89, 95% CI: 0.77 to 1.04, p=0.1443). Another investigation indicated that smoking was associated with reduced all-cause IHM, with only 6.5% of smokers dying during hospitalization compared to 13.2% of non-smokers (OR: 0.46; 95% CI: 0.34-0.63) [39].

The risk of AMI is directly related to the duration and severity of smoking habits, with even indirect exposure to tobacco smoke increasing cardiovascular risk [40,41]. A study by Venkatason et al. found a positive link between smoking and improved outcomes in patients with STEMI and NSTEMI [42]. In the NSTEMI cohort, smokers exhibited a higher incidence of coronary revascularization in the hospital (21.6% for smokers versus 16.7% for non-smokers, P < 0.001). On the contrary, in the STEMI cohort, the rates were comparable between smokers and non-smokers.

 A meta-analysis of PCI trials found that smoking is associated with a higher risk of mortality from all causes and heart failure [43,44]. Furthermore, Gao et al. found an increased risk of recurrent MI associated with smoking [45]. In conclusion, smoking plays a vital role in AMI, affecting its pathogenesis, presentation, treatment, and patient outcomes. Recognizing the harmful impact of tobacco on cardiovascular health emphasizes the need for interventions to stop smoking to reduce morbidity and mortality from CVD and increase its outcome.

Body Mass Index

Obesity, characterized by an elevated BMI, is correlated with an increased probability of cardiovascular diseases such as HTN, dyslipidemia, and DM [46]. Specific research has identified a counterintuitive U-shaped correlation between BMI and long-term cardiovascular health outcomes. This occurrence, known as the "obesity paradox," indicates that individuals with higher BMI could exhibit similar or even reduced mortality rates compared to those with normal BMI. At the same time, those with very low BMI experience poorer outcomes [47]. The obesity paradox likely results from the intricate interplay of various potential mechanisms, including energy reserves, nutritional status, earlier detection of cardiovascular symptoms, faster medical response, and chronic oxidative stress and inflammation [47]. Following are few examples that discuss the importance of BMI as a clinical factor in AMI.

The research carried out by Elbaz-Greener et al. found that hospitalizations for NSTEMI and STEMI were 75.6% and 24.4%, respectively [48]. The same study identified a BMI below 19 kg/m^2^ as an independent predictor of poorer outcomes and IHM through a multivariate analysis (odds ratio (OR): 1.47, 95% confidence interval (CI): 1.29-1.67).

Angerås et al. observed a peak in survival rates among overweight or moderately obese patients (BMI <35), while those who were underweight or had average weight exhibited the highest mortality risk during follow-up periods [49].

In contrast, some studies contradicted the obesity paradox, showing increased mortality rates in patients with a BMI greater than 40 kg/m^2^ [50,51].

Significantly, the underweight group was consistently associated with a significantly elevated risk of IHM in all cardiovascular diseases compared to the standard BMI group (OR: 1.52, 95% CI: 1.45-1.60) [52].

Some studies reported a J-shaped relationship between BMI and mortality in patients hospitalized for AMI in recent years. These results confirm that the "obesity paradox" remains relevant in the contemporary management of AMI [48,53].

The TRACE study indicates that among patients experiencing AMI, overall obesity is inversely correlated with mortality rates. Specifically, patients with an underweight AMI exhibited a higher mortality risk (OR: 1.73; 95% CI 1.23-2.44) [54]. On the contrary, within the same study, overweight women demonstrated a reduced mortality risk (OR: 0.78; 95% CI: 0.68-0.90).

The CRUSADE initiative similarly found that only underweight patients faced an elevated mortality risk (OR: 1.2; 95% CI: 1.0-1.4) [55].

Ellis et al. observed that patients with a BMI of less than 25 kg/m^2^ or more than 35 kg/m^2^ had higher mortality rates after PCI [56].

Powell et al. also identified this bimodal distribution, noting increased mortality risks at both extremes of BMI [57]. Both studies reported a consistent reduction in adverse outcomes for BMI values below 40 kg/m^2^.

Furthermore, the protective nature of obesity has been proposed to be attributable to larger vessel sizes in general [58].

Co-morbidities (Diabetes and HTN)

DM and HTN are critical clinical determinants in the realm of AMI and IHM [59,60]. Over the last four decades, significant advances in outcomes have been documented for the general population with AMI, regardless of the status of the DM. However, consistent observation has shown a two-fold increase in IHM rates among DM patients [60]. In the general population, the incidence of HTN increases progressively with increasing age in both genders; however, it is consistently higher in all age groups in black individuals, posing a more substantial risk factor for coronary artery disease compared to whites. Approximately 54% of Americans aged 65 to 74 years have HTN, whereas the prevalence among black individuals is 72% [61].

The frequency of mortality in hospital settings was markedly higher among hypertensive patients, registering at 5.9% compared to 4.0% (P <0.001) [62]. Research indicated that people with HTN and STEMI are more prone to type 2 diabetes and exhibit elevated blood glucose levels upon admission if they do not have a prior diagnosis of diabetes, which adversely impacts their prognosis [63]. A detailed examination of the Acute Myocardial Infarction Registry (KAMIR), which included data from 8568 Korean patients with STEMI, corroborated that type 2 diabetes is prevalent among patients with hypertensive STEMI. These patients demonstrated poorer clinical and angiographic results, a higher probability of heart failure, and a higher risk of major adverse cardiovascular events (MACE) during long-term follow-up [64]. Sheifer et al. investigated 102,399 patients with AMI and found that diabetes independently predicted delays in treatment initiation (OR: 1.11, 95% CI: 1.07-1.14) [65]. Numerous investigations have indicated that patients with AMI who also have diabetes frequently exhibit non-classical symptoms, such as the absence of persistent chest pain, sweating, and referred pain. A history of diabetes has been identified as an independent predictor of such atypical presentations. These results are consistent with previous studies [66,67].

Before coronary artery bypass graft (CABG) and before percutaneous coronary intervention (PCI)

CABG surgery effectively alleviates symptoms and improves patient outcomes. However, patients who undergo CABG typically exhibit advanced stages of coronary atherosclerosis, predisposing them to an increased risk of symptom recurrence and adverse events [68,69]. In contrast, PCI is a non-surgical method aimed at improving coronary blood flow at the site of obstruction through techniques such as balloon inflation, stent deployment, and/or atherectomy performed via a coronary catheter [70]. The anatomical complexity and blood supply of the coronary artery in patients with a history of CABG differ significantly from those without previous bypass surgery. This complexity complicates the identification of the culprit vessels during emergency angiography, and bypass grafts often complicate the scenario [71]. In a retrospective study by Liu et al., primary PCI procedures in 78 patients with AMI and previous CABG surgery showed a lower success rate and higher IHM compared to patients without previous bypass surgery [71]. The mortality rate within the hospital was found to be 4.6 times higher compared to the study counterparts during the equivalent period of time.

In another observational analysis by Blachutzik et al., findings indicated that of 121 patients with prior CABG had 13% IHM, adverse outcomes were related to advanced age and congestive heart failure [72]. Another investigation, the CAMI registry study that spans 2013-2014, found that 8% of Chinese patients admitted for AMI, including STEMI and NSTEMI with previous CABG and PCI, had a history of previous MI [73]. A study by Xie et al. demonstrated that CABG produced better results compared to PCI IHM (OR= 1.41, 95% CI 1.22-1.63, p< 0.001) [74]. The same research reported that the cumulative meta-analysis of all-cause mortality indicated significant differences between CABG and PCI at three years of follow-up, with the disparity becoming notable at five years of cardiac-specific mortality.

Cardiac troponin and creatine kinase-MB as biomarkers

High-sensitivity cardiac troponin T (hs-cTnT) and creatine kinase MB (CK-MB) are widely recognized biomarkers for the clinical diagnosis of AMI [75]. Measuring cardiac troponin T (cTn-T) in blood is fundamental for identifying MI. As cTnI and cTnT are present exclusively in cardiomyocytes and possess a distinctive cardiac-specific amino acid sequence, they have become the primary biomarkers for the detection of MI or other myocardial injuries [76]. The guideline-recommended approach to diagnosing MI involves measuring cardiac troponin I (cTnI) or cTnT in the blood [77]. The leading cause of MI is inadequate oxygen supply and acute ischemia of cardiac tissue [77]. However, these biomarkers have some limitations, which include sampling over time, poorly predicted long-term outcomes, and unstable angina [78].

The onset of acute ischemia initiates cardiomyocyte necrosis, which causes the breakdown of cell membranes and organelles, leading to the subsequent release of cellular proteins into the bloodstream. This process results in a significant elevation in cTn levels in the blood, typically peaks within 10-20 hours in patients who undergo reperfusion of blocked coronary arteries or 24-50 hours in those without reperfusion after the onset of acute ischemia [79,80]. Elevated troponin levels can persist for up to 10-14 days after MI, much longer than other markers of MI, such as creatine kinase-MB or myoglobin. It is hypothesized that this extended release of cTnI and cTnT from the tissue is due to the binding of the troponin complex to other elements of thin filaments within cardiomyocytes [79,80].

Numerous investigations have shown that these markers can predict immediate and long-term mortality in AMI and are related to the severity of coronary lesions and the size of the infarct [81-83]. As the estimated glomerular filtration rate (eGFR) decreases, the probability of cardiovascular death increases progressively, with approximately half of patients with advanced chronic kidney disease (CKD). In a study involving 5022 patients, 70.5% were diagnosed with STEMI, while 29.5% had NSTEMI [83]. The same research indicated that as eGFR decreased, high-sensitivity cardiac troponin T (hs-cTnT) and IHM increased. At the same time, creatine kinase-MB (CK-MB) did not show a proportional increase, leading to an elevated cTnT/CK-MB ratio. Chronic kidney disease (CKD), a critical prognostic factor in AMI, also affects troponin T levels [84]. A recent meta-analysis highlighted the high sensitivity of hs-cTnT in predicting mortality among patients with CKD, noting that each 10 ng/L increase in hs-cTnT is associated with a 14% increase in the risk of all-cause mortality [85]. The ratio of hs-cTnT to CK-MB also shows promise as a tool for risk stratification in people with AMI and CKD [83]. In an additional study by Sivarajah et al. involving 420 patients, the primary outcome of IHM was significantly higher in the group with elevated cTn levels (14.6% vs. 6.3%; p = 0.008) [86].

CK-MB demonstrates a clinical sensitivity of 90% for the diagnosis of AMI, but its relatively low specificity counterbalances this. It is detectable within 12 hours after the onset of AMI symptoms, reaches peak serum concentrations at 24 to 36 hours, and normalizes in 48 to 72 hours. Due to these release kinetics, total CK measurement is inadequate for the early diagnosis of AMI in six hours [87]. To improve the cardiac-specific diagnosis of AMI, measuring both total CK and CK-MB is suggested, the latter being the cardiac-specific isoenzyme of CK. A CK-MB to CK ratio greater than 6% indicates myocardial injury, while a ratio less than 6% suggests skeletal muscle damage or other non-cardiac causes [87]. Elevated levels of CK-MB in STEMI patients undergoing primary angioplasty are associated with higher mortality rates; however, the relationship between these values and short-term outcomes remains undetermined [88].

Research by Rakowski et al. indicated that the level of CK-MB measured 12 hours after PCI emerged as a more accurate predictor of infarct size at six months compared to levels measured 6, 18, 24, and 48 hours after PCI. This measurement was also more reliable than the CK-MB area under the curve and the peak CK-MB values [89]. Furthermore, CK-MB levels significantly correlate with IHM rates in patients with STEMI who underwent PCI [90]. According to research by Dohi et al., analysis of peak CK-MB levels may be an effective method to estimate the infarct size and predict left ventricular dysfunction. In particular, a peak level of CK-MB that exceeds 300 U/L can be expected to result in around 80% of patients having a large STEMI (infarct size ≥ 17%) following early reperfusion therapy [91].

## Conclusions

Acute management of MI is a complex task that requires understanding and amalgamating numerous clinical factors to improve patient outcomes. In individuals with AMI, clinical variables such as age, sex, smoking status, BMI, comorbid conditions, history of CABG, and PCI, along with biomarkers, affect presentation, prognosis, and therapeutic approaches. Due to physiological changes associated with aging, gender, BMI, smoking, and an increased burden of comorbidities, these clinical factors play an important role in the outcomes of AMI. Patients with HTN, DM, and CKD find it more challenging to manage AMI and have a worse prognosis because they increase myocardial damage. Customized management strategies and comprehensive risk assessments are essential for patients with AMI to optimize outcomes and lower mortality rates.

Patients with DM and HTN are at risk of AMI because they have comorbidity conditions and require personalized treatment approaches. The history of previous CABG or PCI plays a crucial clinical role in AMI, where overall outcomes are affected. A personalized treatment plan and specialized medical attention are necessary to improve results and minimize hospital mortality. Managing AMI in individuals with a history of CABG or PCI requires close collaboration between cardiologists, cardiac surgeons, and multidisciplinary teams. Management of IM relies on biomarkers to diagnose, prognostic, and assess risk. The IHM of patients with AMI is reduced by using biomarker-based methods to quickly detect high-risk individuals. AMI risk prediction and personalized care can be advanced through ongoing research and enhancement of these clinical factors.
